# Maternal bisphenol and phthalate urine concentrations and weight gain during pregnancy

**DOI:** 10.1016/j.envint.2019.105342

**Published:** 2019-12-18

**Authors:** Elise M. Philips, Susana Santos, Eric A.P. Steegers, Alexandros G. Asimakopoulos, Kurunthachalam Kannan, Leonardo Trasande, Vincent W.V. Jaddoe

**Affiliations:** a The Generation R Study Group, Erasmus MC, University Medical Center Rotterdam, Rotterdam, the Netherlands; b Department of Pediatrics, Erasmus MC - Sophia Children’s Hospital, University Medical Center Rotterdam, Rotterdam, the Netherlands; c Department of Obstetrics & Gynaecology, Erasmus MC, University Medical Center Rotterdam, Rotterdam, the Netherlands; d Wadsworth Center, New York State Department of Health, and Department of Environmental Health Sciences, School of Public Health, State University of New York at Albany, Albany NY12201, United States; e Department of Chemistry, the Norwegian University of Science and Technology (NTNU), 7491 Trondheim, Norway; f Biochemistry Department, Faculty of Science, King Abdulaziz University, Jeddah, Saudi Arabia; g Department of Pediatrics, New York University School of Medicine, New York City, New York, United States; h Department of Environmental Medicine, New York University School of Medicine, New York City, New York, United States; i Department of Population Health, New York University School of Medicine, New York City, New York, United States; j New York Wagner School of Public Service, New York City, New York, United States; k New York University Global Institute of Public Health, New York City, New York, United States

**Keywords:** Bisphenol, Phthalate, Gestational weight gain, Cohort studies

## Abstract

**Background:**

Insufficient or excessive gestational weight gain are associated with increased risks of adverse birth and childhood outcomes. Increasing evidence suggests that exposure to bisphenols and phthalates may disrupt hormonal pathways and thereby influence gestational weight gain.

**Objective:**

To examine the associations of early and mid-pregnancy bisphenol and phthalate urine concentrations with gestational weight gain.

**Methods:**

In a population-based prospective cohort study among 1,213 pregnant women, we measured early and mid-pregnancy bisphenol and phthalate urine concentrations. Maternal anthropometrics before pregnancy were obtained by questionnaire and repeatedly measured at our research center during pregnancy. We used linear and logistic regressions to evaluate the associations of bisphenols and phthalates with total and period-specific gestational weight gain.

**Results:**

Higher maternal total bisphenols and bisphenol S were associated with a lower total gestational weight gain at nominal level. Stratification by body mass index group showed that higher total bisphenols and bisphenol S were associated with lower total gestational weight gain specifically in normal weight women (respectively −509 g [95% CI −819, −198] and −398 g [95% CI −627, −169]). Each log unit increase in early pregnancy total bisphenol and bisphenol A urine concentrations were associated with lower mid- to late pregnancy gestational weight gain in the whole group (effect estimates −218 g/log unit increase [95% CI −334, −102] and −132 g/log unit increase [95% CI −231, −34], respectively). These associations were independent of mid-pregnancy compounds. Mid-pregnancy bisphenols and phthalates concentrations were not associated with gestational weight gain.

**Discussion:**

Higher maternal bisphenol urine concentrations in early pregnancy may lead to reduced gestational weight in second half of pregnancy. Further research is needed to assess the effects of maternal bisphenols and phthalates urine concentrations on placental and fetal growth and development.

## 1. Background

Insufficient or excessive gestational weight gain are associated with increased risks of adverse birth and childhood outcomes. The US Institute of Medicine and others have established criteria for excessive as well as insufficient gestational weight gain, recognizing a substantial literature documenting increases in adverse pregnancy, birth and offspring outcomes among women with excessive and insufficient gestational weight gain (Gaillard et al., 2013; Hrolfsdottir et al., 2015; Kaimura et al., 2017; Marchi et al., 2015; Santos et al., 2019).

Gestational weight gain is a multifactorial phenotype. Risk factors for excessive gestational weight gain include nulliparity, higher total energy intake and smoking during pregnancy (Gaillard et al., 2013; Nohr et al., 2008). Studies reporting associations of increased maternal progesterone and leptin levels with greater gestational weight gain suggest that hormonal responses may be important mechanisms contributing to insufficient or excessive gestational weight gain (Lacroix et al., 2016; Lof et al., 2009). A substantial literature has suggested that synthetic chemicals, such as bisphenols and phthalates, can disrupt hormones and thereby influence gestational weight gain (Buser et al., 2014; Cantonwine et al., 2014; Harley et al., 2013; Philips et al., 2017; Trasande et al., 2012; Valvi et al., 2015). For example, mono-ethyl phthalate (MEP) has been associated with lower maternal progesterone levels in the second trimester of pregnancy (Johns et al., 2015). Higher maternal progesterone levels have been associated with increased gestational weight gain (Lof et al., 2009). A study in mice reported increased leptin concentrations in pregnant mice exposed to bisphenol A (BPA) (Alonso-Magdalena et al., 2010). Exposure to bisphenols and phthalates can be modified through behavioral modifications as well as regulatory action (Carwile et al., 2009; Carwile et al., 2011; Harley et al., 2016; Rudel et al., 2011). To our knowledge, the associations of bisphenol and phthalate concentrations with maternal gestational weight gain have not been studied yet.

We examined among 1,213 women participating in a population-based prospective cohort study the associations of early and mid-pregnancy bisphenol and phthalate urine concentrations with total and period-specific gestational weight gain and the risks of insufficient or excessive gestational weight gain.

## 2. Methods

### 2.1. Study design and population for analysis

**Fig. 1.**
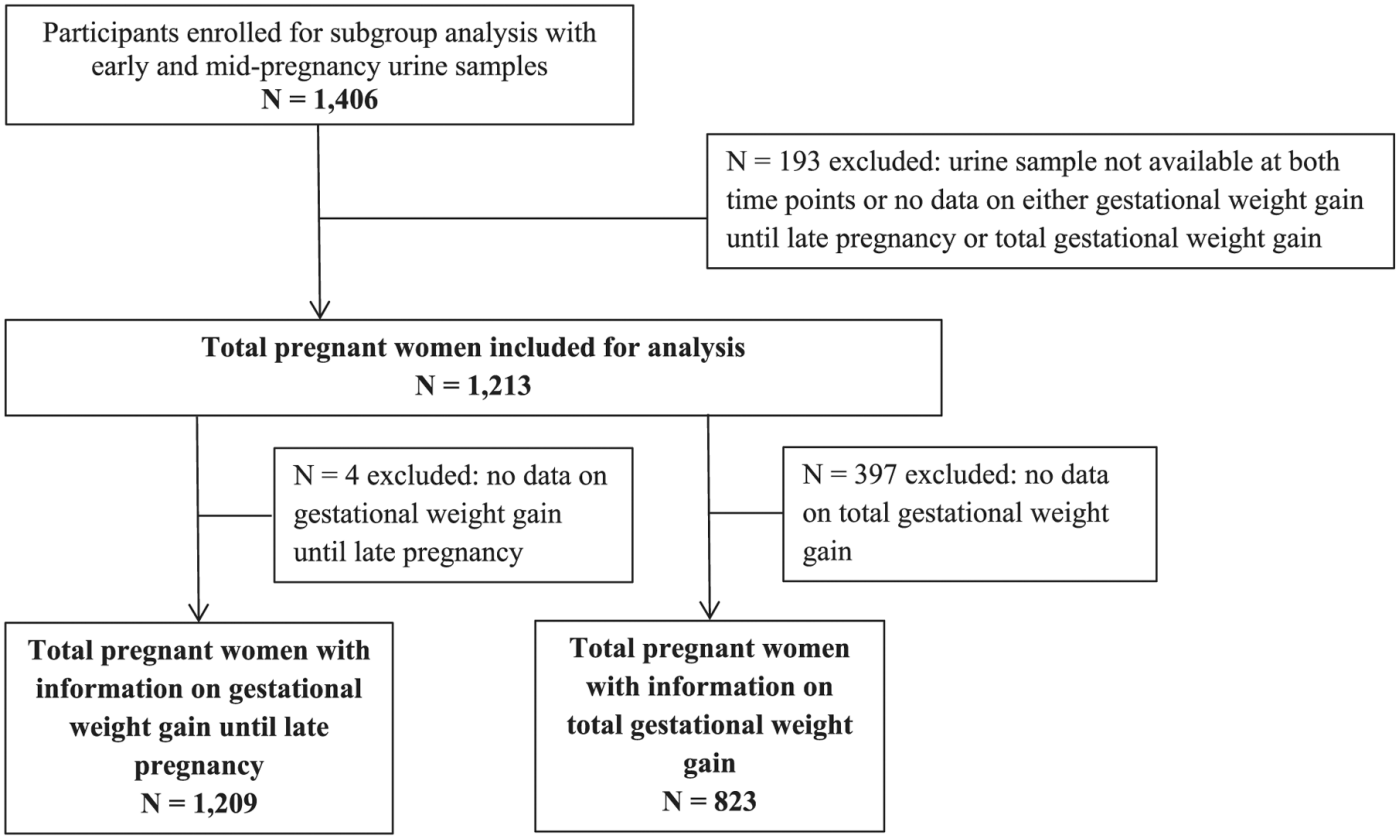
Flowchart.

The present study was embedded in the Generation R Study, a population-based prospective cohort study from early pregnancy onwards (Kooijman et al., 2016). In total, 8,879 women were enrolled in pregnancy, of which 76% before a gestational age of 18 weeks. The study has been approved by the Medical Ethical Committee of the Erasmus Medical Center in Rotterdam. Written consent was obtained from all participating women (World Medical Association 2013). Bisphenol and phthalate urine concentrations were measured in a subgroup study among 1,406 mothers with an available early or mid-pregnancy urine sample and whose children participated in postnatal studies. This subgroup included singleton pregnancies only. We excluded women without an available urine sample at both time points, without information on gestational weight gain until late pregnancy or total gestational weight gain (n = 193), which led to 1,213 women included in the analysis. For analysis on total gestational weight gain and clinical gestational weight gain categories, we excluded women without information on total gestational weight gain (n = 397), leading to 823 women included in those analyses (Fig. 1).

### 2.2. Bisphenol and phthalate urine concentrations

As previously described, bisphenol and phthalate concentrations were measured in a spot urine sample obtained from each subject during the early and mid-pregnancy measurement (median gestational age 13.1 weeks [inter-quartile range (IQR) 12.1–14.5 weeks] and 20.4 weeks [IQR 19.9–20.9], respectively). All urine samples were collected between February 2004 and October 2005. Details on collection, transportation and analysis methodology are provided elsewhere (Philips et al., 2018).

We grouped urinary biomarkers for exposure to phthalates according to their use in product categories. These product categories were first personal care products, and second plasticizers to impart flexibility to plastics. Based on these categories, phthalates we grouped in low and high molecular weight phthalates. We calculated the weighted molar sums for low molecular weight (LMW) phthalate, high molecular weight (HMW) phthalate, di-2-ethylhexylphthalate (DEHP) metabolites, and di-n-octylphthalate metabolites. Phthalic acid (PA) was used separately as a proxy of total phthalate exposure. Among HMW phthalates, DEHP is of particular interest because of its widespread use in food packaging (Serrano et al., 2014). DNOP is also of concern because, although banned from use in the European Union since 2005, its primary metabolite, mono(3-carboxypropyl)phthalate (mCPP), is still detectable in biosamples (Casas et al., 2011; Philips et al., 2018). Individual compounds were included in if they were detected in ≥20% of the samples. Also, bisphenols that were detected in ≥50% of the samples were analyzed separately. For bisphenol and phthalate concentrations below the level of detection we substituted the level of detection divided by the square root of 2, as routinely performed in bisphenols and phthalates (Hornung and Reed 1990). Table 1 shows the metabolites that were included in all separate groups, their values and detection rates.

### 2.3. Maternal anthropometrics

Maternal height (cm) and weight (kg) were measured at enrollment without shoes and heavy clothing and body mass index (kg/m^2^) was calculated. Weight was measured repeatedly during subsequent visits at the research center (early pregnancy median gestational age 13.1 weeks [IQR 12.1, 14.5], mid pregnancy median 20.4 weeks [IQR 19.9, 20.9], and late pregnancy median 30.2 weeks [IQR 29.9, 30.8]). Information on maternal weight just before pregnancy was obtained by questionnaire. In our population for analysis, 68.2% of all women were enrolled before a gestational of 14 weeks. Information on total weight during pregnancy was assessed by questionnaire 2 months after delivery (median gestational age at delivery 40.3 [IQR 39.3, 41.0]). Total gestational weight gain was calculated as the difference between the highest weight before birth and pre-pregnancy weight and was available in a subgroup of 823 mothers. For sensitivity analysis, gestational weight gain until the late pregnancy visit was calculated as the difference between late pregnancy weight and pre-pregnancy weight and was available for 1,209 mothers. Correlation of late pregnancy weight and total weight was 0.96 (P-value < 0.001).

According to the IOM guidelines, we classified total gestational weight gain as insufficient, sufficient and excessive in relation to maternal pre-pregnancy BMI (Institute of Medicine and National Research Council Committee to Reexamine IOM Pregnancy Weight Guidelines 2009). Weight gain was further analyzed in specific periods of pregnancy (weight gain between the measured weight at the early and mid-pregnancy visit; weight gain between the measured weight at the mid- and late pregnancy visit; and weight gain between the measured weight at the late pregnancy visit and reported total pregnancy weight).

### 2.4. Covariates

Covariates were selected based on previous analyses of potential determinants of first trimester bisphenol and phthalate concentrations (Philips et al., 2018). Information on maternal age at enrollment, educational level, ethnicity, parity, pre-pregnancy weight, and folic acid supplementation use was obtained from the first questionnaire at enrollment. Information on smoking and alcohol consumption was assessed by questionnaires in each trimester (Jaddoe et al., 2010). Maternal daily dietary intake was assessed at enrollment using a modified version of the validated semi-quantitative food-frequency questionnaire (FFQ) of Klipstein-Grobusch et al. (Klipstein-Grobusch et al., 1998). The FFQ covered the average dietary intake over the previous three months, covering the dietary intake in the first trimester of pregnancy (Tielemans et al., 2016). We used caloric intake derived from the FFQ as a covariate in statistical analyses.

### 2.5. Statistical analysis

Differences in subject characteristics between groups of gestational weight gain were assessed using one-way ANOVA tests for continuous variables and chi-square tests for proportions. Non-response analysis was performed to assess distributions of maternal characteristics and investigated outcomes. For the main analyses, all bisphenol and phthalate urinary metabolite concentrations were log-transformed to account for right skewness in the distribution.

We performed multivariable linear and multinomial logistic regressions to evaluate associations of early and mid-pregnancy urinary concentrations with total gestational weight gain continuously, gestational weight gain per pregnancy period and clinical categories of gestational weight gain. To investigate total gestational weight gain continuously and in clinical categories, early and mid-pregnancy bisphenol and phthalate groupings were used simultaneously to examine the relative influence of early versus mid-pregnancy urinary concentrations. When testing associations of gestational weight gain in specific pregnancy periods, metabolite concentrations of all earlier time points were added simultaneously to the model to adjust for measures at other visits. Therefore, models for early-to-mid-pregnancy gestational weight gain included metabolite concentrations in early pregnancy only. Because detection rates of bisphenol S (BPS) dropped below 50% in mid-pregnancy, early pregnancy BPS concentrations were adjusted for total bisphenol concentrations in mid-pregnancy.

For all significant models, subanalyses of individual bisphenol compounds or phthalate metabolites were performed to determine which metabolites were driving the association. Subanalysis of significant models with early and mid-pregnancy concentrations of bisphenols and phthalates used simultaneously were performed with the separate compounds of the significant group together with the total group of the other pregnancy period, to keep models comparable. As a sensitivity analysis, we used multivariable linear regression models to examine the associations between the logs of molar concentrations of the metabolite groups with gestational weight gain until late pregnancy.

In all models, urinary concentrations of each bisphenol or phthalate compound or grouping were converted to μg/g or μmol/g creatinine to adjust for dilution (Barr et al., 2005). All models were adjusted for maternal age, educational level, ethnicity, parity, daily dietary caloric intake, folic acid supplement use, smoking, and alcohol consumption. Higher pre-pregnancy BMI has been associated with a lower gestational weight gain (Santos et al., 2018). Our previous studies showed that higher pre-pregnancy BMI was associated with higher bisphenol and phthalate concentrations in early pregnancy (Philips et al., 2018). Therefore, models with gestational weight gain as outcome were additionally adjusted for pre-pregnancy BMI. To investigate potential effect modification by pre-pregnancy BMI of the associations of bisphenol and phthalate concentrations with gestational weight gain, we have tested interaction terms with categories of pre-pregnancy BMI. Additionally stratified analyses have been performed for significant interactions. Non-linear effects of early and mid-pregnancy metabolite concentrations on total gestational weight gain were assessed using quartiles.

Missing data of the covariates were imputed using multiple imputation. Five imputed data sets were created. Effect estimates were pooled to obtain the overall result, taking into account the within and between imputation variance according to Rubin’s Rules (Rubin 1987). The percentage of missing values within the population for analysis were lower than or equal to 10%, except for maternal folic acid supplementation use (17.0%) and daily dietary caloric intake (23.8%). To correct for multiple hypothesis testing, each p-value was compared with a threshold defined as 0.05 divided by the effective number of independent tests estimated based on the correlation between the exposures (p-value threshold of 0.011) (Li et al., 2012). All analyses were performed using the Statistical Package of Social Sciences version 21.0 for Windows (SPSS Inc, Chicago, IL, USA).

## 3. Results

### 3.1. Subject characteristics

Mid-pregnancy urine concentrations of bisphenols and phthalates were generally lower than in early pregnancy. Also, detection rates of BPS, bisphenol F (BPF), mono-hexylphthalate (mHxP) and mono-2-heptylphthalate (mHpP) urine concentrations were considerably lower in mid-pregnancy (Table 1). Characteristics of the included mothers are given in Table 2. Of all women, 19.1%, 30.0%, and 50.9% had insufficient, sufficient, and excessive gestational weight gain, respectively. Women with excessive gestational weight gain had a higher pre-pregnancy BMI and were more often younger, smokers, and nulliparous. As shown in Supplementary Table S1, nonresponse analysis showed similar distributions of sociodemographic factors and other risk factors for gestational weight gain in the subgroup study population as in the entire study cohort. However, the subgroup of mothers with information on total gestational weight gain tended to be slightly higher educated and healthier.

### 3.2. Bisphenol and phthalate urine concentrations and gestational weight gain

Early and mid-pregnancy phthalates were not associated with total gestational weight gain (Table 3). For total bisphenols and BPS, associations with a decreased total gestational weight gain were observed at nominal level.

We observed effect modification by pre-pregnancy BMI of the associations of bisphenol and phthalate concentrations with total gestational weight gain (statistical interaction *p-value* < 0.1) for early pregnancy total bisphenols, BPA, BPS, PA, LMW phthalate metabolites and DEHP metabolites (*data not shown*). Further stratification yielded significant results for total bisphenols and BPS in the normal weight group with a decreased total gestational weight gain (respectively −509 g (95% CI −819, −198) and −398 g (95% CI −627, −169), both p-value = 0.001). To illustrate, an interquartile range increase in total bisphenols was associated with −864 g (95% CI −1391, −336) decrease in total gestational weight gain among normal weight women. Because the numbers per stratum were low for underweight and obese women these analyses were not presented as main analyses. Assessment of potential non-linear association of early and mid-pregnancy bisphenol and phthalate concentrations using quartiles did not reveal any indications of non-linearity (*data not shown*).

Each log unit increase in early pregnancy total bisphenol urine concentrations was associated with −218 g (95% CI −334, −102) gestational weight gain in mid- to late pregnancy. Analysis of individual bisphenol compounds in early pregnancy showed that maternal BPA concentrations were driving this association with a −132 g (95% CI −231, −34) lower mid- to late pregnancy weight gain/log unit increase. The associations of early pregnancy BPS and BPF urine concentrations with gestational weight gain in mid-to-late pregnancy tended toward nominal significance (Table 3 and Supplementary Table S2). Early pregnancy DNOP metabolite concentrations were associated with mid- to late pregnancy weight gain at nominal level. Bisphenol and phthalate concentrations in early and mid-pregnancy were not associated with early-to-mid-pregnancy weight gain or late pregnancy-to-total gestational weight gain. We did not observe effect modification by pre-pregnancy BMI for the analyses on gestational weight gain during specific periods of pregnancy (*data not shown*).

### 3.3. Bisphenol and phthalate levels and clinical categories of gestational weight gain

Table 4 shows that bisphenol and phthalate urine concentrations in early and mid-pregnancy were not associated with insufficient or excessive gestational weight gain. Early pregnancy LMW phthalate metabolites were associated with higher odds of insufficient gestational weight gain, However, this associations attenuated into non-significance correction for multiple testing.

### 3.4. Sensitivity analysis

Sensitivity analysis shows that the associations of early pregnancy bisphenols with gestational weight gain until late pregnancy somewhat attenuated but had the same directionality (Supplementary Table S3). Early pregnancy BPS concentrations were associated with gestational weight gain until late pregnancy at nominal level, adjusted for total bisphenol concentrations in mid-pregnancy. However, this associations attenuated into non-significance after correction for multiple testing.

## 4. Discussion

Results from this prospective population-based cohort study showed that among normal weight women total gestational weight gain was lower for women with higher total bisphenols or BPS concentrations in early pregnancy, independent of bisphenol concentrations in mid-pregnancy. Early pregnancy total bisphenols and BPA were associated with a lower gestational weight gain in mid- to late pregnancy in the whole group.

### 4.1. Interpretation of main findings

To the best of our knowledge, this is the first prospective study that examined the associations of maternal bisphenols and phthalates concentrations with gestational weight gain. Our findings suggest that maternal bisphenol concentrations in early pregnancy are associated with a lower gestational weight gain, mainly in second half of pregnancy. Additionally, the findings suggest that women with a normal weight are most vulnerable for effects of early pregnancy bisphenols on gestational weight gain. We did not observe associations of bisphenol and phthalate concentrations with clinical categories of gestational weight gain. An additional analysis suggests that among women with insufficient weight gain each log unit increase in total bisphenols was associated with a stronger reduction in weight gain than in women with sufficient and excessive weight gain (*data not shown*). Since we have only used early and mid-pregnancy bisphenol and phthalate urine concentrations, we cannot rule out that also late pregnancy bisphenol and phthalate concentrations have a certain effect on gestational weight gain. However, this seems unlikely, since the associations of early pregnancy exposures were independent of mid pregnancy exposure concentrations.

Previous cross-sectional studies investigating determinants of bisphenols and phthalates reported associations of higher concentrations of BPA and phthalates in pregnant women with a higher BMI (Arbuckle et al., 2014; Cantonwine et al., 2014; Lewin et al., 2017; Philips et al., 2018; Valvi et al., 2015). A recent prospective study of pregnant women reported a negative association between DEHP metabolites in early pregnancy and early gestational weight gain (Bellavia et al., 2017). Persistent organic pollutants (POPs) have also been examined for associations with gestational weight gain. Similar to bisphenols and phthalates, the majority of POPs are lipophilic chemicals, except for perfluoroalkyl substances (PFASs) (DeWitt 2015; Stockholm Convention). The results from studies investigating effects of POPs on gestational weight gain show different associations with gestational weight gain for the PFASs and other POPs. Higher perfluorooctanesulfonate (PFOS) levels – a perfluoroalkyl substance - before and in early pregnancy have been associated with a higher gestational weight gain in normal and underweight women, while in overweight women this effect was not observed (Ashley-Martin et al., 2016; Jaacks et al., 2016). Other POPs, including dichlorodiphenyl dichloroethene (DDE), polychlorinated bisphenyls (PCBs) in early pregnancy and neonatal DDE, hexachlorocyclohexanes (HCHs), PCBs and polybrominated diphenyl ethers (PBDEs), have been associated with lower or even insufficient gestational weight gain (Herbstman et al., 2007; Vafeiadi et al., 2014; Vizcaino et al., 2014). Thus our study results add to previous studies suggesting that various environmental exposures in specifically early pregnancy may influence gestational weight gain.

Gestational weight gain is a complex phenotype (Gaillard et al., 2013; Nohr et al., 2008). Besides increased maternal fat storage, several compartments could be responsible for the observed change in gestational weight gain. Information about maternal fat storage, measurements of body composition during pregnancy would be informative. However, measurements of body composition during pregnancy were not available in the current study. In our previous study, we did not observe associations of early pregnancy bisphenol and phthalate concentrations with placental weight at birth (Philips et al., 2019). A previous study within the same cohort suggested lower fetal growth in association with maternal BPA concentrations (Snijder et al., 2013). Gestational weight gain, in particular in mid- and late pregnancy, is associated with birth weight (Ay et al., 2009; Gaillard et al. 2013). In a recent rodent study, early pregnancy BPA exposure was associated with impaired remodeling of the uterine spiral arteries and intrauterine growth restriction (Muller et al., 2018). Altogether, previous studies and our results suggest that higher maternal bisphenol urine concentrations in early pregnancy may lead to reduced gestational weight in second half of pregnancy. Further research is needed to assess the effects of maternal bisphenol and phthalate urine concentrations on different aspects of gestational weight gain, such as placental and fetal growth and development.

### 4.2. Strengths and limitations

Strengths of this study were the prospective data collection from early pregnancy onwards, large sample size of 1,213 participants with a urine sample in early and mid-pregnancy, and information on gestational weight gain. The subgroup of women with information on total gestational weight gain tended to a slightly higher educated, healthier population, which might have influenced results. However, sensitivity analysis of gestational weight gain until late pregnancy and period-specific gestational weight gain argue against biased estimates. The response rate at baseline was 61% (Kooijman et al., 2016). Although we cannot rule out selection towards a relatively healthy population, selection bias in cohort studies is more likely to arise from loss to follow up rather than from non-response at baseline (Nohr et al., 2006). Additionally, models have been adjusted for several potential proxies for health, reducing the odds of biased estimates due to selection bias. Less variation in our study population than in the general population may have led to underestimation of effect estimates. Repeated exposures were analyzed using multiple regression analysis, enabling investigation of potential windows of vulnerability (Chen et al., 2015). In our analysis, collinearity was not an issue (Supplementary Table S4). Bisphenol and phthalate metabolites were measured in spot urine samples in early and mid-pregnancy and typically have half-lives of less than 24 h (Braun et al., 2013; Mattison et al., 2014). A single spot urine sample for phthalates could reasonably reflect exposure for up to three months (Hauser et al. 2004), but bisphenols have a high temporal variability, even over the day (Vernet et al., 2019). This non-differential misclassification is expected to lead to attenuation bias in dose-response relationships.

A common method to account for dilution of urinary chemical concentrations is via creatinine adjustment (O’Brien et al., 2016). Endogenous creatinine clearance, measured by 24-hr urine collection, remains the most precise estimation of the glomerular filtration rate in pregnant women (Ahmed et al., 2009). A recent study suggested that specific gravity adjustment is a better correction method in pregnant women (MacPherson et al., 2018). Unfortunately, specific gravity measurements were not available. Additional analysis of models without creatinine adjustment yielded similar results (*data not shown*).

Maternal weight was measured during the visits at our research center. Information on maternal pre-pregnancy weight and total weight during pregnancy was self-reported. Self-reported weight tends to be underestimated, leading to misclassification. Consequently, this might have led to biased estimates. In the period-specific analysis, early-to-mid and mid-to-late pregnancy analyses were based on measured weights only and provide therefore the most reliable estimates. Detailed information on a large number of potential confounding factors was available. Nonetheless, due to the observational design of the study, residual confounding due to unmeasured environmental exposures, socio-demographic or lifestyle factors still might still be an issue.

### 4.3. Conclusion

Higher maternal bisphenol urine concentrations in early pregnancy may lead to reduced gestational weight in second half of pregnancy. Further research is needed to assess the effects of maternal bisphenols urine concentrations on placental and fetal growth and development.

## Supplementary Material

Supp Mat

## Figures and Tables

**Table 1:** Bisphenol and phthalate urinary concentrations (n = 1,213).

	Early pregnancy (< 18 weeks) Median (IQR) (ng/mL)	Percentage of values below the limit of detection (LOD)	Mid-pregnancy (18–25 weeks) Median (IQR) (ng/mL)	Percentage of values below the limit of detection (LOD)
**Total bisphenols**^1^	9.31 (3.61, 20.85)		6.31 (3.04, 13.87)	
Bisphenol A (BPA)	1.67 (0.71, 3.61)	21.2	1.46 (0.74, 3.19)	6.7
Bisphenol S (BPS)	0.35 (0.17, 1.09)	31.9	0.24 (0.12, 0.49)	70.9
Bisphenol F (BPF)	0.58 (0.30, 1.31)	59.6	NA	88.5
**Phthalic acid (PA) metabolites**	57.38 (31.03, 123.45)	0.3	149.68 (61.74, 280.94)	0.1
**Low molecular weight (LMW) metabolites**^1^	1080.01 (425.05, 2940.32)		586.77 (238.87, 1444.95)	
Monomethylphthalate (mMP)	5.59 (2.75, 9.85)	0.2	3.47 (1.84, 6.21)	0.2
Monoethylphthalate (mEP)	136.55 (41.15, 488.49)	0.1	72.64 (25.05, 222.41)	-
Mono-isobutylphthalate (mIBP)	20.93 (9.52, 45.65)	0.2	8.88 (4.59, 17.80)	-
Mono-n-butylphthalate (mBP)	16.08 (7.01, 30.94)	0.7	9.68 (5.51, 18.91)	-
**High molecular weight (HMW) metabolites**^1^	219.09 (112.60, 403.22)		131.83 (73.85, 242.94)	
***Di-2-ethylhexylphthalate (DEHP) metabolites***^1^	171.59 (89.23, 323.30)		96.82 (53.12, 183.72)	
Mono-(2-ethyl-5-carboxypentyl)phthalate (mECPP)	16.09 (8.25, 31.29)	0.2	10.45 (5.77, 19.98)	0.1
Mono-(2-ethyl-5-hydroxyhexyl)phthalate (mEHHP)	11.84 (5.76, 22.80)	0.2	5.57 (2.96, 10.68)	0.1
Mono-(2-ethyl-5-oxohexyl)phthalate (mEOHP)	7.75 (3.54, 15.34)	0.1	7.44 (3.68, 16.30)	-
Mono-[(2-carboxymethyl)hexyl]phthalate (mCMHP)	14.06 (7.60, 26.36)	0.1	4.02 (2.27, 7.38)	0.2
***Di-n-octylphthalate (DNOP)***	5.78 (3.17, 10.81)		3.53 (2.06, 6.77)	
Mono(3-carboxypropyl)phthalate (mCPP)	1.45 (0.80, 2.71)	0.2	0.89 (0.52, 1.70)	0.1
***Other high molecular weight metabolites***				
Monobenzylphthalate (mBzP)	6.40 (3.06, 12.55)	8.0	5.27 (2.29, 11.19)	1.5
Mono-hexylphthalate (mHxP)	0.33 (0.16, 0.62)	23.9	NA	98.7
Mono-2-heptylphthalate (mHpP)	1.09 (0.58, 2.33)	35.4	NA	96.8

NA: not applicable; bisphenol or phthalate is not included in the group due to > 80% below the limit of detection.

1 Groups are molar concentrations in nmol/L with non-detectable levels of separate metabolites imputed as LOD/sqr(2). Separate metabolites are included only if < 80% of values was below the LOD.

**Table 2:** Subject characteristics by gestational weight gain classification^1^

	Total	Insufficient gestational weight gain	Sufficient gestational weight gain	Excessive gestational weight gain	p-value^2^
	n = 1,213	n = 157	n = 247	n = 419	
Maternal age (years)	30.6 (4.8)	31.2 (4.5)	31.8 (4.1)	30.6 (4.6)	0.005
Pre-pregnancy BMI (kg/m^2^)*	22.7 (20.8, 25.3)	22.1 (20.7, 23.9)	21.9 (20.2, 23.7)	23.2 (21.0, 25.7)	0.000
Educational level					0.020
Low	583 (48.1)	70 (45.2)	85 (34.7)	187 (45.3)	
High	596 (49.1)	85 (54.8)	160 (65.3)	226 (54.7)	
Missings	35 (2.8)	–	–	–	
Ethnicity					0.751
Dutch/European	756 (62.3)	107 (69.0)	176 (71.3)	302 (72.2)	
Non-European	452 (37.3)	48 (31.0)	71 (28.7)	116 (27.8)	
Missings	5 (0.4)	–	–	–	
Parity					0.001
Nulliparous	742 (61.2)	85 (54.1)	152 (61.5)	293 (69.9)	
Multiparous	471 (38.8)	72 (45.9)	95 (38.5)	126 (30.1)	
Missings	–	–	–	–	
Daily dietary caloric intake (kcal)	2080 (5 0 8)	2035 (5 4 8)	2145 (4 9 0)	2113 (4 8 5)	0.163
Creatinine early pregnancy (< 18 weeks) μg/mL)*	1030 (491, 1661)	958 (522, 1582)	1026 (450, 1705)	1032 (472, 1642)	0.647
Creatinine mid-pregnancy (18–25 weeks) μg/mL)*	1164 (740, 1818)	1291 (814, 2025)	1222 (676, 1861)	1106 (709, 1654)	0.375
Smoking					0.000
Never	867 (71.5)	136 (86.6)	192 (77.7)	286 (68.3)	
Until pregnancy was known	108 (8.9)	7 (4.5)	19 (7.7)	50 (11.9)	
Continued	160 (13.2)	7 (4.5)	21 (8.5)	57 (13.6)	
Missings	78 (6.4)	7 (4.5)	15 (6.1)	26 (6.2)	
Alcohol consumption					0.566
Never	491 (40.5)	64 (40.8)	84 (34.0)	157 (37.5)	
Until pregnancy was known	193 (15.9)	24 (15.3)	47 (19.0)	62 (14.8)	
Continued	451 (37.2)	63 (40.1)	101 (40.9)	172 (41.1)	
Missings	78 (6.4)	6 (3.8)	15 (6.1)	28 (6.7)	
Folic acid supplementation					0.893
No	191 (15.7)	18 (11.5)	28 (11.3)	51 (12.2)	
Start first 10 weeks	330 (27.2)	40 (25.5)	62 (25.1)	122 (29.1)	
Start periconceptional	484 (39.9)	72 (45.9)	116 (47.0)	189 (45.1)	
Missings	208 (17.1)	27 (17.2)	41 (16.6)	57 (13.6)	
Early to-mid pregnancy weight gain (kg)*	3.0 (2.0, 5.0)	–	–	–	
Mid- to late pregnancy weight gain (kg)*	5.0 (3.5, 7.0)	–	–	–	
Late to total pregnancy weight gain (kg)*	4.5 (3.0, 7.0)	–	–	–	
Total gestational weight gain(kg)*	15.0 (12.0, 18.0)	–	–	–	
Gestational weight gain until late pregnancy (kg)*	10.0 (8.0, 13.0)	–	–	–	

1 Values are means (standard deviation) or numbers of subjects (percentage). Only women with available information on total gestational weight gain were classified in a total gestational weight gain category (n = 823).

2 Differences between groups of insufficient, sufficient and excessive gestational weight gain were assessed using one-way ANOVA tests for continuous variables and chi-square tests for proportions.

* Median (IQR range)

**Table 3:** Associations of early and mid-pregnancy bisphenol and phthalate urine concentrations with gestational weight gain (n = 1,213).

	Gestational weight gain (grams) Early to mid-pregnancy, (95% Confidence Interval) (n = 1,205)	Mid- to late pregnancy, (95% Confidence Interval) (n = 1,207)^1^	Late pregnancy to total, (95% Confidence Interval) (n = 819)^1^	Total (95% Confidence Interval) (n = 823)^1^
**Early pregnancy (< 18 weeks)**				
Total bisphenols	0 (−98, 98)	−218 (−334, −102) * ^†^	−82 (−261, 98)	−354 (−641, −68)*
Bisphenol A	17 (−66, 100)	−132 (−231, −34)* ^†^	−54 (−205, 98)	−125 (−367, 117)
Bisphenol S^2^	−26 (−97, 44)	−76 (−160, 7)	−41 (−169, 87)	−261 (−466, −56)*
Phthalic acid	32 (−84, 147)	−139 (−277, 0)	−131 (−334, 71)	−50 (−375, 274)
LMW phthalate metabolites	63 (−33, 159)	−110 (−230, 9)	−196 (−375, −17)*	−191 (−478, 96)
HMW phthalate metabolites	13 (−113, 140)	−133 (−285, 18)	−175 (−411, 61)	−268 (−646, 111)
DEHP metabolites	24 (−100, 147)	−122 (−270, 27)	−183 (−413, 47)	−259 (−627, 109)
DNOP metabolites	40 (−83, 162)	−176 (−324, −29)*	−218 (−436, −1)*	−319 (−666, 29)
**Mid-pregnancy (18**-**25 weeks)**				
Total bisphenols	–	−119 (−251, 14)	161 (−35, 356)	143 (−168, 453)
Bisphenol A	–	−112 (−238, 14)	151 (−36, 338)	147 (−150, 444)
Bisphenol S	–	–	–	–
Phthalic acid	–	−125 (−271, 21)	217 (−6, 440)	33 (−323, 389)
LMW phthalate metabolites	–	−86 (−221, 49)	145 (−56, 346)	60 (−262, 381)
HMW phthalate metabolites	–	−149 (−304, 5)	112 (−129, 353)	−30 (−415, 354)
DEHP metabolites	–	−140 (−292, 11)	156 (−81, 393)	64 (−315, 444)
DNOP metabolites	–	−68 (−235, 99)	96 (−149, 342)	−112 (−505, 280)

Estimates are based on multivariate regression analyses. Increases are per log unit increase in early and mid-pregnancy urinary Total bisphenols/BPA/BPS/Phthalic acid/LMW/HMW/DEHP/DNOP metabolite concentrations per gram creatinine. All models are adjusted for maternal age, maternal pre-pregnancy BMI, daily dietary caloric intake, parity, ethnicity, education, maternal smoking, maternal alcohol, and folic acid supplementation. In total, 1,213 women are included in the analyses in this table. Due to random nonresponse, not all women had available information about all the weights.

1 Early and mid-pregnancy compounds have been used in the model simultaneously, yielding estimates adjusted for compounds at the other time point.

2 For models of early pregnancy BPS, the total group of mid-pregnancy bisphenols has been used in the model simultaneously, if applicable. Estimates for mid-pregnancy total bisphenols in these models are not presented.

* p-value < 0.05

† significant after multiple testing correction

**Table 4:** Associations of early and mid-pregnancy bisphenol and phthalate urine concentrations with clinical categories of gestational weight gain (n = 823).

	Insufficient weight gain, Odds Ratio (95% Confidence Interval) (n = 157)	Excessive weight gain, Odds Ratio (95% Confidence Interval) (n = 419)
**Early pregnancy (< 18 weeks)**		
Total bisphenols	0.96 (0.82, 1.13)	0.91 (0.80, 1.03)
Bisphenol A	0.97 (0.84, 1.11)	0.96 (0.86, 1.07)
Bisphenol S^1^	1.03 (0.92, 1.15)	0.96 (0.88, 1.06)
Phthalic acid	1.18 (0.98, 1.41)	1.11 (0.96, 1.28)
LMW phthalate metabolites	1.18 (1.01, 1.39)*	1.03 (0.91, 1.17)
HMW phthalate metabolites	1.16 (0.94, 1.43)	1.00 (0.84, 1.18)
DEHP metabolites	1.16 (0.94, 1.42)	1.00 (0.85, 1.18)
DNOP metabolites	1.15 (0.95, 1.40)	0.97 (0.83, 1.13)
**Mid pregnancy (18**-**25 weeks)**		
Total bisphenols	0.94 (0.79, 1.12)	1.02 (0.89, 1.17)
Bisphenol A	0.94 (0.79, 1.11)	1.03 (0.90, 1.17)
Bisphenol S	–	–
Phthalic acid	0.97 (0.80, 1.19)	1.00 (0.86, 1.17)
LMW phthalate metabolites	0.97 (0.81, 1.16)	0.97 (0.84, 1.12)
HMW phthalate metabolites	0.92 (0.75, 1.14)	0.97 (0.82, 1.14)
DEHP metabolites	0.91 (0.74, 1.12)	0.98 (0.83, 1.15)
DNOP metabolites	0.96 (0.78, 1.19)	0.88 (0.74, 1.04)

Estimates are based on multivariate regression analyses. Reference category is sufficient weight gain. Only women with available information on total gestational weight gain were classified in a total gestational weight gain category. Increases are per log unit increase in early and mid-pregnancy urinary total bisphenols/BPA/BPS/Phthalic acid/LMW/HMW/DEHP/DNOP metabolite concentrations per gram creatinine. Models are adjusted for maternal age, daily dietary caloric intake, parity, ethnicity, education, maternal smoking, maternal alcohol, and folic acid supplementation. Early and mid-pregnancy compounds have been used in the model simultaneously, yielding estimates adjusted for compounds at the other time point.

1 For models of early pregnancy BPS, the total group of mid-pregnancy bisphenols has been used in the model simultaneously, if applicable. Estimates for second trimester total bisphenols in these models are not presented.

* p-value < 0.05

## Abbreviations:

BPA: bisphenol A
BPF: bisphenol F
BPS: bisphenol S
DDE: dichlorodiphenyl dichloroethene
DEHP: Di-2-ethylhexylphthalate
DNOP: di-n-octylphthalate
FFQ: food-frequency questionnaire
GWG: gestational weight gain
HCHs: hexachlorocyclohexanes
HMW: high molecular weight
IQR: inter-quartile range
LMW: low molecular weight
mCPP: mono)3-carboxypropyl) phthalate
MEP: mono-ethyl phthalate
mHpP: mono-2-heptylphthalate
mHxP: mono-hexylphthalate
PA: phthalic acid
PBDEs: polybrominated diphenyl ethers
PCBs: polychlorinated bisphenyls
PFASs: perfluoroalkyl substances
PFOS: perfluorooctanesulfonate
POPs: persistant organic pollutions
