# Supplementary material for: Maternal bisphenol and phthalate urine concentrations and weight gain during pregnancy
Source: Environ Int. Author manuscript; Available in PMC 2021 Aug 4. (PMC8336629; doi:10.1016/j.envint.2019.105342)
Supplement: Supp Mat [file NIHMS1718262-supplement-Supp_Mat.docx]

**Supplemental Material**

**Maternal bisphenol and phthalate urine concentrations**

**and weight gain during pregnancy**

Elise M. Philips et al.

**Supplementary Table S1.** Non-response analysis.

**Supplementary Table S2.** Subanalysis of associations of early pregnancy bisphenol F with mid- to late gestational weight gain

**Supplementary Table S3.** Associations of early and mid-pregnancy bisphenol and phthalate urine concentrations with gestational weight gain until late pregnancy

**Supplementary Table S4.** Within and between correlations for early and mid-pregnancy compounds

**Supplementary Table S1. Nonresponse analysis^1^**

|  | **Sample n= 823**  **Information on maximum gestational weight gain** | **Sample n = 1,213 Information on maximum gestational weight gain or gestational weight gain until late pregnancy** | **Sample n = 3,927**  **Sample from original cohort not included in subgroup study on maternal bisphenol and phthalate urine concentrations** |
| --- | --- | --- | --- |
| Maternal pre-pregnancy BMI (kg/m^2) 2^ | 22.4 (20.7, 24.8) | 22.7 (20.8, 25.3) | 22.6 (20.8, 25.3) |
| Maximum GWG (kg) | 15.0 (5.6) | 15.0 (5.6) | 15.0 (5.8) |
| GWG until late pregnancy (kg) | 10.5 (4.3) | 10.4 (4.7) | 10.5 (4.8) |
| IOM classification |  |  |  |
| Insufficient weight gain | 19.1 | 19.1 | 18.4 |
| Sufficient weight gain | 30.0 | 30.0 | 30.4 |
| Excessive weight gain | 50.9 | 50.9 | 51.3 |
| Maternal age (y) | 31.0 (4.5) | 30.6 (4.8) | 30.4 (4.9) |
| Daily dietary caloric intake | 2109 (499) | 2080 (508) | 2054 (544) |
| Parity (% nulliparous) | 64.4 | 61.2 | 59.9 |
| Ethnicity (% Dutch/European) | 71.3 | 62.6 | 64.7 |
| Education (% high) | 58.0 | 50.6 | 48.8 |
| Maternal smoking (% nonsmoking) | 79.2 | 76.4 | 73.8 |
| Maternal alcohol use (% no alcohol) | 39.4 | 43.3 | 42.4 |
| Maternal folic acid supplement use (% periconceptional) | 54.0 | 48.2 | 45.6 |

^1^Values represent means (standard deviation) or valid percentages
^2^Median (IQR)

**Supplementary Table S2. Subanalysis of associations of early pregnancy bisphenol F with mid- to late gestational weight gain**

|  | **Gestational weight gain**  **mid- to late pregnancy (grams)**  **(95% Confidence Interval)**  **(n=1,207)** |
| --- | --- |
| Bisphenol F | -116 (-234, 1) |

Estimates are based on multivariate regression analyses. Increases are per log unit increase in early pregnancy urinary BPF concentrations per gram creatinine, adjusted for mid-pregnancy total bisphenols concentration. Models are adjusted for maternal age, maternal pre-pregnancy BMI, daily dietary caloric intake, parity, ethnicity, education, maternal smoking, maternal alcohol, and folic acid supplementation.

*p-value<0.05

**Supplementary Table S3. Associations of early and mid-pregnancy bisphenol and phthalate urine concentrations with gestational weight gain until late pregnancy (n=1,209)**

|  | **Gestational weight gain until late pregnancy (grams)**  **(95% Confidence Interval)** |
| --- | --- |
| **Early pregnancy (<18 weeks)** |  |
| Total bisphenols | -159 (-354, 36) |
| Bisphenol A | -47 (-212, 119) |
| Bisphenol S^1^ | -142 (-282, -2)* |
| Phthalic acid | 32 (-200, 265) |
| LMW phthalate metabolites | 26 (-174, 226) |
| HMW phthalate metabolites | -52 (-306, 203) |
| DEHP metabolites | -13 (-261, 235) |
| DNOP metabolites | -101 (-348, 147) |
| **Mid-pregnancy (18-25 weeks)** |  |
| Total bisphenols | -79 (-302, 144) |
| Bisphenol A | -58 (-270, 155) |
| Bisphenol S | - |
| Phthalic acid | -217 (-462, 28) |
| LMW phthalate metabolites | -59 (-286, 168) |
| HMW phthalate metabolites | -127 (-386, 131) |
| DEHP metabolites | -52 (-306, 202) |
| DNOP metabolites | -261 (-541, 19) |

Estimates are based on multivariate regression analyses. Increases are per log unit increase in early and mid-pregnancy urinary total bisphenols/BPA/BPS/Phthalic acid/LMW/HMW/DEHP/DNOP metabolite concentrations per gram creatinine. Models are adjusted for maternal age, maternal pre-pregnancy BMI, daily dietary caloric intake, parity, ethnicity, education, maternal smoking, maternal alcohol, and folic acid supplementation. Early and mid-pregnancy compounds have been used in the model simultaneously, yielding estimates adjusted for compounds at the other time point. ^1^For models of early pregnancy BPS, the total group of mid-pregnancy bisphenols has been used in the model simultaneously, if applicable. Estimates for second trimester total bisphenols in these models are not presented.

*p-value<0.05

**Supplementary Table S4. Within and between correlations for early and mid-pregnancy compounds (n=1,213)**

|  | Mid-pregnancy | |  |  |  |  |  |  |
| --- | --- | --- | --- | --- | --- | --- | --- | --- |
| Early pregnancy | Total bisphenols | Bisphenol A | Bisphenol S | Phthalic Acid | LMW phthalate metabolites | HMW phthalate metabolites | DEHP phthalate metabolites | DNOP phthalate metabolites |
| Total bisphenols | **0.027** | 0.988*† | - | 0.254*† | 0.085* | 0.250*† | 0.265*† | 0.232*† |
| Bisphenol A | 0.829*† | **0.064*** | - | 0.240*† | 0.078* | 0.242*† | 0.255*† | 0.231*† |
| Bisphenol S | 0.489*† | 0.192*† | - | - | - | - | - | - |
| Phthalic Acid | 0.340*† | 0.314*† | 0.228*† | **0.128*†** | 0.583*† | 0.534*† | 0.527*† | 0.516*† |
| LMW phthalate metabolites | 0.202*† | 0.185*† | 0.147*† | 0.701*† | **0.325*†** | 0.315*† | 0.281*† | 0.286*† |
| HMW phthalate metabolites | 0.340*† | 0.307*† | 0.258*† | 0.546*† | 0.404*† | **0.119*†** | 0.942*† | 0.669*† |
| DEHP phthalate metabolites | 0.335*† | 0.303*† | 0.254*† | 0.532*† | 0.376*† | 0.979*† | **0.104*†** | 0.647*† |
| DNOP phthalate metabolites | 0.299*† | 0.254*† | 0.277*† | 0.512*† | 0.366*† | 0.735*† | 0.710*† | **0.180*†** |

Spearman’s correlation coefficients for early and mid-pregnancy compounds and compound groups, used as µg/g or µmol/g creatinine. Correlations of compounds or compound groups within early pregnancy are displayed in darker grey. Correlations of compounds or compound groups within mid-pregnancy are displayed in light grey. Correlations of compounds between the pregnancy periods are bold.
Bisphenol S for mid-pregnancy is excluded for this table, as is for the total paper, because of a low detection rate.
